# EBV reactivation and immunoparalysis indicate a harmful immune endotype in sepsis

**DOI:** 10.1186/s13054-026-05966-2

**Published:** 2026-05-07

**Authors:** K. S. Rosiewicz, M. Anft, U. Stervbo, S. Skrzypczyk, S. Kaliszczyk, B. Koos, K. Rump, H. Nowak, M. Unterberg, T. H. Westhoff, T. Brenner, M. Trilling, M. Schmueck-Henneresse, K. Wolk, R. Sabat, J. Gregorius, F. Wappler, A. Zarbock, M. Adamzik, N. Babel

**Affiliations:** 1 Center for Translational Medicine, Medical Department 1, Marien Hospital Herne, University Hospitals of the Ruhr-University of Bochum, Hölkeskampring 40, 44625 Herne, Germany; 2 Center for Regenerative Therapies, BCRT, Berlin Institute of Health, Charité-Universitätsmedizin Berlin, Augustenburger Platz 1, 13353 Berlin, Germany; 3 Klinik für Anästhesiologie, Intensivmedizin und Schmerztherapie, Knappschaft Kliniken Universitätsklinikum Bochum GmbH, Ruhr Universität Bochum, In der Schornau 23-25, 44892 Bochum, Germany; 4 Marien Hospital Herne, University Hospitals of the Ruhr-University of Bochum, Medizinische Klinik 1, Hölkeskampring 40, 44625 Herne, Germany; 5 Department of Anesthesiology and Intensive Care Medicine, University Hospital Essen, University Duisburg-Essen, Hufelandtsr. 55, 45147 Essen, Germany; 6 Institute for Virology, University Hospital Essen, University of Duisburg-Essen, Essen, Germany; 7 Institute for the Research on HIV and AIDS-associated Diseases, University Hospital Essen, University of Duisburg-Essen, Essen, Germany; 8 Klinik für Dermatologie, Venerologie und Allergologie, Charité - Universitätsmedizin Berlin, Campus Charité Mitte, Luisenstraße 2, 10117 Berlin, Germany; 9 Klinik für Anästhesiologie und operative Intensivmedizin, Klinikum Köln-Merheim, Ostmerheimer Str. 200, 51109 Köln, Germany; 10 Klinik und Poliklinik für Anästhesiologie und operative Intensivmedizin, Universitätsklinikum Münster, Albert-Schweitzer-Str. 33, 48149 Münster, Germany

## Abstract

**Background:**

Sepsis is increasingly recognized as a highly dynamic immunological disorder in which hyperinflammation and anti-inflammatory processes occur simultaneously. The clinical phenotype depends on which arm predominates at a given time, resulting in an early phase that is typically dominated by hyperinflammation and a subsequent phase characterized by hypoinflammation, also referred to as immunoparalysis (IP). Epstein-Barr virus (EBV) reactivation has been associated with an immunosuppressive status. However, its interaction with IP and resulting immune phenotypes remains poorly defined so far. In this current study, we investigated the temporal dynamics of EBV reactivation and IP status, and assessed their impact on immune signatures and mortality in sepsis.

**Methods:**

In this retrospective cohort of 124 intensive care unit (ICU) patients with sepsis, we performed analysis of EBV load by qPCR and analyzed the inflammatory stage by quantifying HLA-DR molecules on monocytes (mHLA-DR) using flow cytometry. Patients with < 5,000 mHLA-DR/monocyte were classified positive for immunoparalysis (IP+). Cytokine profiles and vital sign were analyzed in parallel. Patients were assigned to four sepsis groups based on EBV/IP status (EBV- IP-, EBV + IP-, EBV- IP+, EBV + IP+). Time-dependent Cox models (start-stop structure) were used to estimate hazard ratios (HR) for mortality, adjusted for age, sex and Sequential Organ Failure Assessment (SOFA) score. Cytokines and clinical markers were compared using Kruskal-Wallis and rank-based analyses.

**Results:**

EBV positivity was associated with higher hazard of death (HR 3.30, 95% CI 1.24–8.81, *p* = 0.0009), while IP showed a similar but nonsignificant trend (HR 2.14, 95% CI 0.93–4.90, *p* = 0.073). The combined EBV + IP+ group exhibited the highest mortality (HR 7.23, 95% CI 2.24–23.3, *p* = 0.0009). This group also showed the strongest cytokine activation (IL-6, IL-8, IL-10, IL-17 A, IL-18, MCP-1) and lowest mHLA-DR expression, indicating a mixed hyperinflammatory and immunosuppressed phenotype. In contrast, EBV- IP- patients displayed the most immunocompetent baseline profile.

**Conclusion:**

Our preliminary findings suggest that EBV reactivation superimposed on immunoparalysis is associated with a harmful sepsis endotype combining excessive cytokine activity with impaired monocyte function. Further studies are needed to evaluate if the dynamic monitoring of EBV DNA and mHLA-DR expression may enable early identification of patients at highest risk and guide targeted immunomodulatory or antiviral interventions.

**Supplementary Information:**

The online version contains supplementary material available at 10.1186/s13054-026-05966-2.

## Introduction

Sepsis represents a life-threatening organ dysfunction caused by a dysregulated host response to infection and remains one of the leading causes of mortality in intensive care units (ICUs) worldwide. Despite advances in antimicrobial therapy and supportive care, the underlying immune pathology of sepsis continues to challenge clinical management. Rather than occurring as two distinct phases, hyperinflammation and anti-inflammatory responses in sepsis arise in parallel, with clinical phenotype reflecting the dominant immune state at a given time. Early in the diseases course, hyperinflammatory mechanisms often predominate, whereas a shift toward hypoinflammation, marked by lymphocyte apoptosis, monocyte dysfunction, and impaired antigen presentation, may emerge later. This imbalance toward immunosuppression contributes to secondary infections and unfavorable outcomes and has been conceptualized as a sepsis-induced immunoparalysis (IP) [1–3].

A key marker of IP is the expression of human leukocyte antigen-DR (HLA-DR) on circulating monocytes. Reduced monocyte HLA-DR (mHLA-DR) levels correlate strongly with impaired immune responsiveness, increased risk of nosocomial infections, and mortality [4–7]. The threshold of < 5,000 molecules per monocyte, originally proposed by Döcke et al. [8] has been widely adopted as an operational cutoff to define clinically relevant IP [9–11]. Mechanistically, persistent exposure to inflammatory mediators such as IL-6, IL-10, and glucocorticoids contributes to down-regulation of mHLA-DR and disruption of cellular cross-talk [6, 12, 13]. This creates a vulnerable immune landscape prone to viral reactivation and secondary infection.

Among latent viruses, Epstein-Barr virus (EBV) has emerged as an important indicator of immune dysregulation in critically ill patients. EBV reactivation occurs in up to 50–70% of ICU patients and is associated with prolonged ICU stay, higher Sequential Organ Failure Assessment (SOFA) scores, and increased mortality [14–17]. While traditionally regarded as an epiphenomenon of immune exhaustion, accumulating evidence suggests that EBV may actively modulate host immunity once being reactivated. Viral antigens and non-coding RNAs can stimulate nuclear factor kappa-B (NF-κB), Toll-like receptors (TLRs), and inflammasome pathways, leading to enhanced cytokine production including Interleukin-6 (IL-6), Interleukin-8 (IL-8), Interleukin-18 (IL-18), and monocyte chemoattractant protein 1 (MCP-1) [18–22]. Consequently, EBV reactivation may amplify systemic inflammation while simultaneously reflecting an underlying failure of antiviral T- and NK cell control.

This bidirectional relationship between EBV reactivation and host immunosuppression may create a self-perpetuating loop of inflammation and immune exhaustion. In this context, EBV positive septic patients may represent a biological distinct subgroup characterized by mixed antagonistic response syndrome (MARS), a state of concurrent hyperinflammation and immunosuppression [23, 24]. However, previous studies have largely examined viral reactivation or immune paralysis in isolation, without addressing their temporal interplay or combined effect on outcome.

To address this gap, we conducted a retrospective cohort study in 124 critically ill sepsis patients, in whom EBV viral load (by qPCR) and mHLA-DR expression were measured repeatedly during the first week of ICU treatment. By combining these parameters, patients were classified into four immunovirological phenotypes: EBV- IP-, EBV + IP-, EBV- IP+, EBV + IP+. Using a time-dependent Cox model adjusted for age, sex, and SOFA score, we investigated how these dynamic immune states were associated with short-term mortality alongside with respective cytokine profiles and renal and metabolic function parameters. We aimed to delineate specific inflammatory signatures within each subgroup, and identify a certain high-risk sepsis phenotype.

## M&M

### Study and patients

The samples analyzed in this study originated from the SepsisDataNet.NRW cohort. Here, patients were prospectively enrolled at five university hospitals in Germany from March 2018 to October 2019 (German Clinical Trial Registry Nos. DRKS00018871) in accordance with the Sepsis-3 criteria [25]. The exclusion criteria were as follows: (1) individuals younger than 18 years at the time of ICU admission (2), refusal or retraction of consent, and (3) cessation of treatment. Within the SepsisDataNet.NRW study, biomaterials, including serum and peripheral blood mononuclear cells (PBMCs), were collected on days 0, 4, and 7 following study inclusion. Data of demographic characteristics (age, sex, comorbidities) and major outcome at day 30 of ICU stay (death or survival) were collected as well. The presented study represents a retrospective analysis of prospectively collected clinical and immunological data from 152 critically ill sepsis patients. Datasets of 28 patients were excluded from this study due to missing EBV determination qPCR results.

### Screening for EBV

Blood samples collected on ICU days 0, 4, and 7 were screened for EBV DNA by qPCR as described elsewhere [26]. Briefly, DNA was isolated and purified using the QIAamp DNA Blood Mini Kit (Qiagen) and tested for EBV by RealStar EBV Kit 1.0. Baseline EBV serostatus was not routinely available. Given the adult ICU population studied (median age 64 years) and the given EBV seroprevalence (> 90%) in the general adult population in Germany, EBV DNAemia detected during ICU stay was interpreted as being consistent with EBV reactivation rather than primary infection.

### Quantification of monocytic HLA-DR molecules

Whole blood was used for the quantification of HLA-DR expression on monocytes (mHLA-DR) by flow cytometry as previously described [8]. Briefly: Within 4 h after blood collections, whole blood was stained with CD14 (PerCP-Cy5.5, AB_2737726, BD, USA) and HLA-DR (PE, AB_2916858, BD, USA) antibodies. HLA-DR molecules per cell were quantified with BD Quantibrite™PE-Kit (BD, USA) per manufactures’ instruction. Patients with < 5,000 mHLA-DR molecules were classified as immunoparalyzed (IP+).

### Routine diagnostics

Patients were closely and intensively monitored during their stay in the intensive care unit (ICU). The Sepsis-related Organ Failure Assessment (SOFA) score was used to assess the severity of sepsis at the ICU arrival. Kidney, and blood pressure values were analyzed using routine hospital diagnostics.

### Evaluation of pro-inflammatory cytokines

Collected serum samples were analyzed by flow cytometry for pro-inflammatory cytokines using the Biolegend^®^ LEGENDplex™ Human Inflammation Panel 1 kit (13-Plex).

### Statistics

All statistical analyses were performed using RStudio (Vers. 23.09.1, R Vers. 4.3.1). A two-sided *p*-value < 0.05 was considered as statistically significant. Comparisons of continuous variables between the four sepsis groups were performed separately within each predefined observational interval (ICU days 0–4, 4–7, and 7–30), reflecting the time-varying nature of EBV and IP status. Within each interval, global group differences were assessed using the non-parametric Kruskal-Wallis test. When this global test was significant, pairwise comparisons were conducted using Dunn’s post-hoc test with Holm adjustment for multiple testing. No statistical comparison across intervals were performed to avoid mixing dependent and independent observations.

To evaluate overall differences across all repeated measurements while accounting for intra-individual dependency, linear mixed-effects models were applied. Log_10_-transformed marker values were modeled as a function of sepsis group and visit (fixed effects), including a random intercept for patient ID to account for repeated measurements within individuals. Pairwise comparisons were performed using estimated marginal means with Holm adjustment for multiple testing.

Categorial variables were compared using χ^2^ or Fisher’s exact test, as appropriate. Results are presented as medians with interquartile ranges (IQR) or absolute/relative frequencies. Given the exploratory nature of this study, no additional correction for multiple testing across different biomarkers was applied [27, 28].

Because patients could change their EBV or IP status during the ICU stay, survival analyses were conducted using time-dependent Cox proportional hazards models in which EBV/IP group membership was treated as a time-varying exposure rather than an absorbing state. Accordingly, patients could contribute risk time to more than one EBV/IP category across predefined time intervals. A start-stop interval structure was applied (0–4 days, 4–7 days, 7–30 days), reflecting the predefined sampling time points. Patients were censored at the time of death or ICU discharge and did not contribute further risk time thereafter. Hazard ratios (HR) and 95% confidence intervals (CIs) were calculated for each covariate. Models were adjusted for age, sex, and SOFA score (or without SOFA for sensitivity analysis), using cluster-robust standard errors at the patient level to account for repeated measurements and intra-patient correlation.

For descriptive analysis of interval-specific mortality, Poisson regression models were applied. Death events and patient-days at risk were aggregated by time interval and sepsis group. Mortality rates were expressed as events per 100 patient-days with exact 95% Poisson CIs. Global group differences per interval were tested using likelihood ratio tests (Omnibus test).

To identify variables that best discriminated between sepsis groups, mean rank values were calculated for each group and variable. The difference between the highest and the lowest mean rank was used as a descriptive measure of discriminatory power. Results were visualized using a rank-based heatmap (z-standardized mean ranks) and a radar plot displaying normalized mean ranks (scaled 0–1) to illustrate subgroup-specific immune signatures. These analyses were considered exploratory and descriptive.

Missing measurements at individual time points did not results in exclusion of entire patients. All analyses were performed using the available observations, and mixed-effects as well as time-dependent Cox models inherently accommodate unbalanced repeated measurements under a maximum likelihood framework. Thus, patients contributed data from all time points at which measurements were available. Only patients with completely missing EBV qPCR data were excluded, as EBV status represents a central variable of the study.

All visualizations (boxplots, heatmaps, radar plots, forest plots, and survival curves) were generated using RStudio using ggplot2, ggpubr, and fmsb packages.

## Results

### Demographic patient characteristics

Out of 152 critically ill sepsis patients, 124 were included in this retrospective study. 28 patients were excluded due to missing blood samples for EBV qPCR determination. For baseline cohort characteristics, patients were grouped into survivors and non-survivors. Sex (n females / males: survivors 35 / 53 vs. non-survivors 17 / 17; *p* = 0.3), median age (survivors 63 years [± 15.28] vs. non-survivors 65.00 years [± 14.77] ;*p* = 0.8), and EBV reactivation (n negative/positive: survivors 20 / 69 vs. non-survivors 7 / 28; *p* > 0.9 ) showed no statistical differences. Obviously, time to event (survivors, discharge: median days 30; non-survivors, death: median days 7; *p* < 0.0001] and SOFA score (survivors: median score 7.00 [± 3.06]; non-survivors: median score 11.50 [± 3.51]; *p* < 0.0001] were highly significant between survived and deceased patients. Further cohort characteristics are summarized in Table 1.

**Table 1:** Clinical baseline characteristics of the study cohort

Characteristic	Category	Survivors (*n* = 89)	Non-survivors (*n* = 35)	*p*-value	Overall *n* = 124
		median [± SD] or *n*	median [± SD] or *n*		
Time to event	Survivors: discharge; Non-survivors: death	30.00 [± 2.88]	7.00 [± 8.11]	< 0.0001	30.00 [± 9.52]
Age	Years	63.00 [± 15.28]	65.00 [± 14.77]	0.8	64.00 [± 15.08]
SOFA	Score	7.00 [± 3.06]	11.50 [± 3.51]	< 0.001	8.00 [± 3.71]
mHLA-DR	Number of molecules	4,405.44 [± 3,854.41]	4,046.83 [± 3,343.09]	0.085	4,177.94 [± 3,729.07]
Sex	Female Male Unknown	35 (40%) 53 (60%) 1	17 (50%) 17 (50%) 1	0.3	52 (43%) 70 (57%) 2
EBV status	Negative Positive	20 (22%) 69 (78%)	7 (20%) 28 (80%)	> 0.9	27 (22%) 97 (78%)
IP EBV group	EBV- IP- EBV + IP- EBV- IP+ EBV + IP+	24 (27%) 11 (12%) 42 (47%) 12 (13%)	7 (20%) 4 (11%) 16 (46%) 8 (23%)	0.6	31 (25%) 15 (12%) 58 (47%) 20 (16%)
Obesity	No Yes Unknown	54 (68%) 26 (33%) 9	17 (61%) 11 (39%) 7	0.6	71 (66%) 37 (34%) 16
CKD	No Yes Unknown	61 (76%) 19 (24%) 9	22 (79%) 6 (21%) 7	> 0.9	83 (77%) 25 (23%) 16
COPD	No Yes Unknown	68 (85%) 12 (15%) 9	25 (89%) 3 (11%) 7	0.8	93 (86%) 15 (14%) 16
Diabetes	No Yes Unknown	58 (73%) 22 (28%) 9	17 (61%) 11 (39%) 7	0.3	75 (69%) 33 (31%) 16
Dialysis	No Yes Unknown	73 (91%) 7 (8.8%) 9	28 (100%) 0 (0%) 7	0.2	101 (94%) 7 (6.5%) 16
Hypertension	No Yes Unknown	27 (34%) 53 (66%) 9	9 (32%) 19 (68%) 7	> 0.9	36 (33%) 72 (67%) 16
CVD	No Yes Unknown	50 (63%) 30 (38%) 9	19 (68%) 9 (32%) 7	0.7	69 (64%) 39 (36%) 16
Malignant tumor	No Yes Unknown	65 (81%) 15 (19%) 9	20 (71%) 8 (29%) 7	0.3	85 (79%) 23 (21%) 16
Nicotine	No Yes Unknown	62 (78%) 18 (23%) 9	26 (93%) 2 (7.1%) 7	0.092	88 (81%) 20 (19%) 16
Transplant	No Yes Unknown	69 (86%) 11 (14%) 9	24 (86%) 4 (14%) 7	> 0.9	93 (86%) 15 (14%) 16

Statistical comparison was performed by the Mann-Whitney *U* test or Fisher/Chi^2^ test. CKD, chronic kidney disease; COPD, chronic obstructive pulmonary disease; CVD, cardio-vascular disease; mHLA-DR, monocyte HLA-DR; SD, standard deviation

To evaluate potential confounding by antiviral or immunomodulatory therapy, we performed additional sensitivity analyses (Supplementary Table 1). Pre-baseline immunomodulator exposure was documented in 25/124 patients (20%), and antiviral therapy in 13/124 patients (10%). Pre-baseline immunomodulator use was not significantly associated with baseline immunoparalysis (Fisher’s exact test *p* = 0.49; OR 0.70, 95% CI 0.26–1.90), and pre-baseline antiviral therapy was not associated with baseline EBV positivity (*p* > 0.99; OR 1.15, 95% CI 0.24–4.49). We further examined whether ICU-administered therapy influenced subsequent immune or viral dynamics. Among patients without baseline immunoparalysis, ICU-post-day0 immunomodulator exposure was not significantly associated with later IP development (*p* = 0.39; OR 2.13, 95% CI 0.27–15.2). Similarly, ICU-post-day0 antiviral therapy was not associated with subsequent EBV reactivation (*p* > 0.99; OR 1.02, 95% CI 0.05–62.1). Collectively, these analyses do not indicate a major confounding effect of antiviral or immunomodulatory therapy on the observed EBV/IP- mortality association.

### Analysis of EBV reactivation and IP in the whole patient cohort

**Fig. 1:**
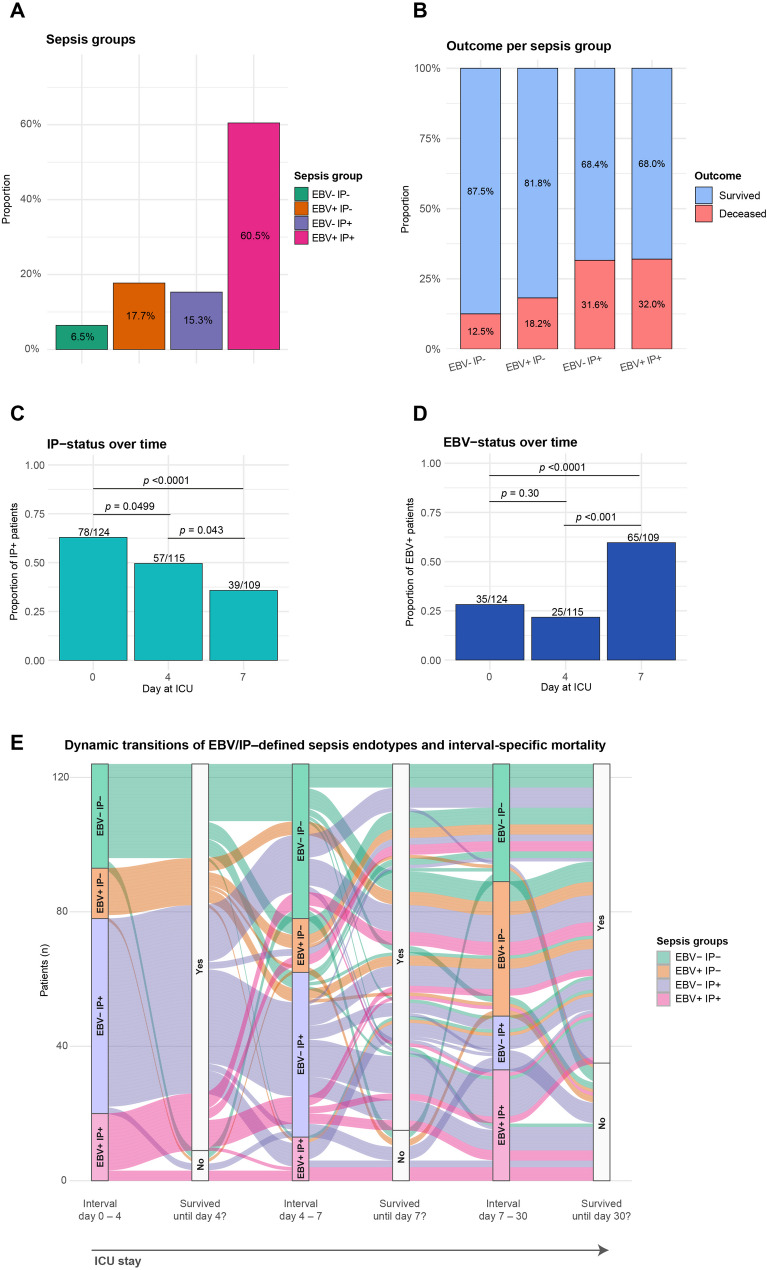
Distribution of sepsis groups and temporal dynamics of immunoparalysis and EBV reactivation. **A** Proportional distribution of the four sepsis groups based on cumulative classification over the entire ICU stay. Patients were assigned to a group if they ever fulfilled the respective criterion during observation (e.g., a patient who became EBV + at any time was classified as EBV+), independent of when the status change occurred. **B** Clinical outcome (survival vs. death) within the four sepsis groups defined in (A). The proportion of deceased patients was markedly higher in both IP+ groups compared to IP- groups. **C** Proportion of patients with immunoparalysis (IP+) at each sampling day. This reflects the actual immune status per day, showing a gradual decline in IP+ prevalence over time. **D** Proportion of patients with EBV reactivations (EBV+) at each sampling day. Here, the prevalence of EBV+ patients increased markedly over time, with significant rise from day 4 to day 7, indicating progressive viral reactivation during the ICU course. Also, this analysis reflects the actual EBV status per day which was not accounted for in (A) and (B). Statistical comparison in C + D by Fisher’s exact test. **E** Alluvial plot showing patient-level transitions between EBV/IP – defined sepsis endotypes across the intervals 0–4, 4–7, and 7–30 days after ICU admission. Flows are colored according to baseline endotype and represent individual patients. Flow width reflects patient number. Mortality status is displayed at day 4, 7, and 30 based on recorded time of death (Survived No = deceased; Survived Yes = survived)

We first categorized patients into four sepsis subgroups according to their EBV and IP characteristics, independent of the timepoint of detection: EBV- IP- (*n* = 8, 6.5%); EBV + IP- (*n* = 22, 17.7%); EBV- IP+ (*n* = 19, 15.3%); EBV + IP+ (*n* = 75, 60.5%) (Fig. 1A). This cumulative classification assigned patients to a positive group (e.g. EBV + or IP+) if they ever fulfilled the respective criterion during ICU stay, including transient mHLA-DR values below 5,000 molecules. Although survival proportions did not differ statistically between the groups, both IP+ groups showed nearly a two-fold higher proportion of deaths (Fig. 1B). Interestingly, while the proportion of IP+ patients decreased from day 0 to day 7, the number of EBV+ patients increased over the same period (Fig. 1C + D). However, we observed substantial intra-patient dynamics in endotype assignments over time. To illustrate this dynamic nature of EBV/IP-defined sepsis endotypes, an alluvial plot was generated showing patient-level transitions across three predefined time intervals (Fig. 1E). The plot reveals considerable endotype switching during ICU stay and highlights that mortality events occurred across dynamic trajectories rather than within static subgroup classifications.

### EBV reactivation and IP positivity are associated with increased mortality in a time-dependent manner

To assess whether EBV and IP status were time-dependently associated with outcome, our first intuitive thought was to perform a Kaplan-Meier survival curve analysis. However, this cumulative approach does not account for dynamic changes in EBV or IP status over time, nor for the fact that patients may transition between groups over time. Classifying patients based on post-baseline status (e.g. ever EBV-positive), would therefore misattribute person-time and potentially introduce immortal-time bias. Therefore, a time-dependent Cox proportional hazards model was applied, in which EBV and IP status were re-evaluated at each pre-defined interval (0–4 days; 4–7 days; 7–30 days). Group membership was updated accordingly for each interval, and patients contributed risk time only until death or discharge.

**Fig. 2:**
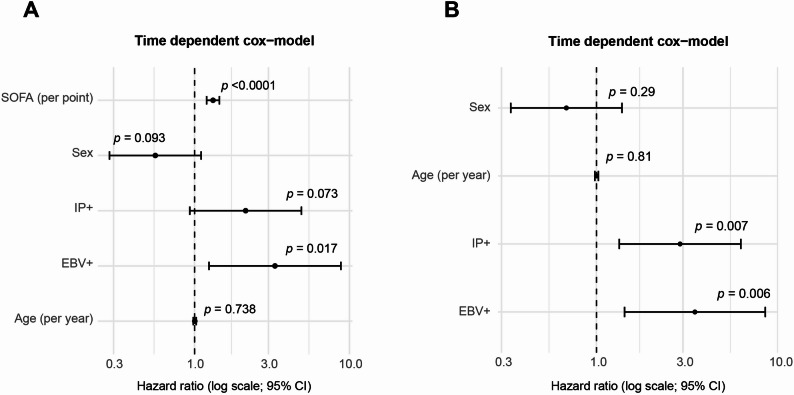
Time-dependent Cox models assessing the effect of EBV reactivation and immunoparalysis on mortality. **A** Time-dependent Cox model including SOFA score, EBV reactivation (EBV+), immunoparalysis (IP+), age and sex. Higher SOFA scores were strongly associated with increased mortality, while EBV positivity also predicted higher risk. **B** Corresponding model excluding SOFA score to isolate the effects of EBV and IP. In the model without SOFA score, immunoparalysis was associated with increased mortality risk. This association was attenuated after adjustment for disease severity (SOFA score). Both models were based on start-stop interval data to account for the time-dependent status of EBV and IP at each measurement point. Hazard ratios (HRs, log scale) are displayed with 95% robust confidence intervals, using patient ID as cluster variable. *p*-values are indicated within the graphs

In the primary time-dependent Cox model adjusted for SOFA score, age, and sex, EBV positivity was significantly associated with higher mortality (HR 3.30, 95% CI 1.24–8.81; *p* = 0.017), while IP positivity showed a similar but borderline effect (HR 2.14, 95% CI 0.93–4.9; *p* = 0.0713; Fig. 2A). As expected, the SOFA score was the strongest predictor of death (HR 1.32, 95% CI 1.20–1.45; *p* < 0.0001), whereas age and sex were not independently associated with outcome. To further explore the relationship between EBV reactivation, immunoparalysis, and mortality we performed an exploratory sensitivity anlysis excluding the SOFA score. This model was used to assess the extend to which the observed associations might be influenced by overall disease severity. In this model, EBV positivity remained significantly associated with mortality (HR 3.49, 95% CI 1.43–8.53, *p* = 0.006). Immunoparalysis was also associated with increased mortality risk (HR 2.89, 95% CI 1.34–6.26, *p* = 0.007; Fig. 2B). As shown in the fully adjusted model including SOFA (Fig. 2A), the association between immunoparalysis and mortality was attenuated, indicating that this effect is at least partly related to disease severity.

**Fig. 3:**
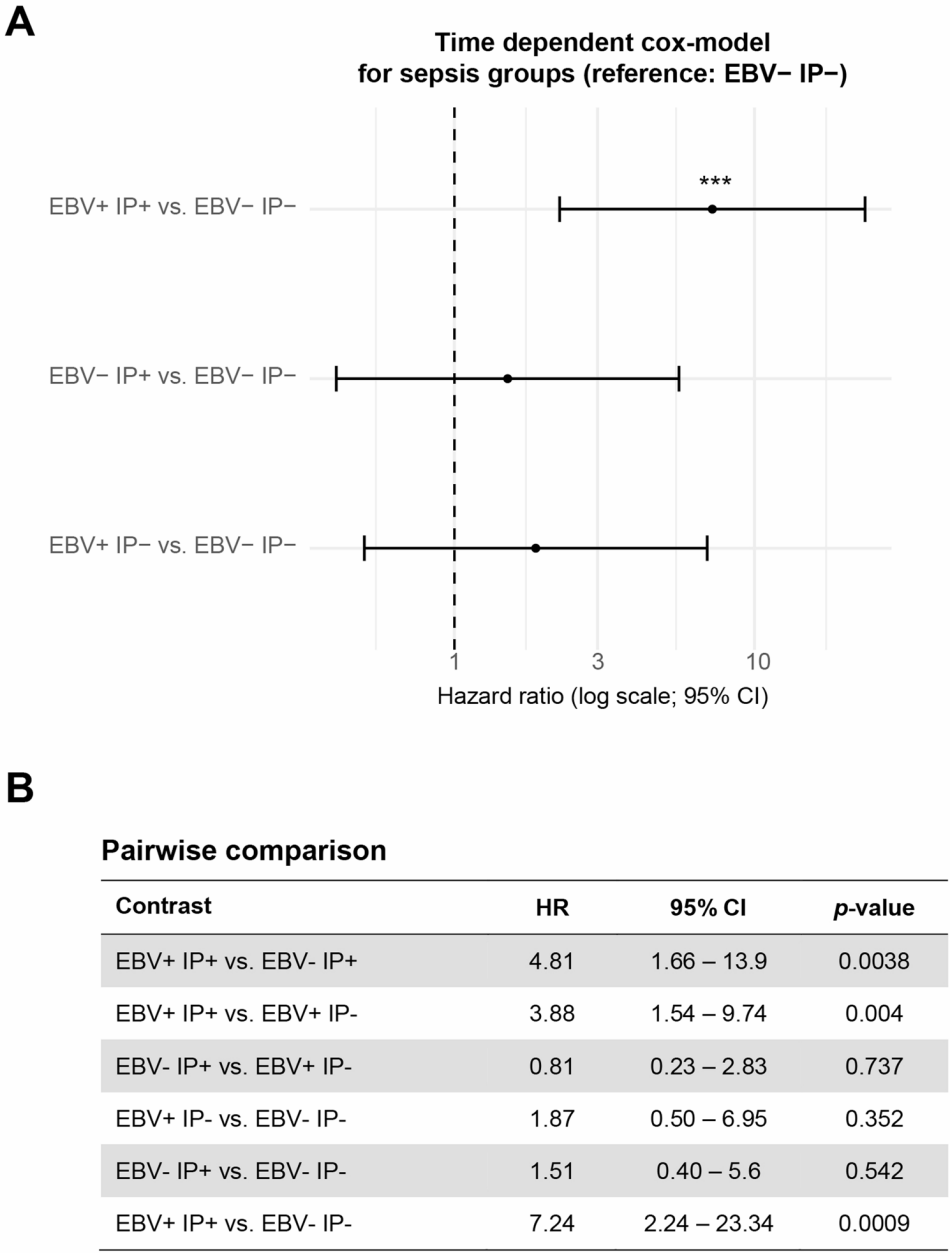
Time-dependent Cox model for sepsis groups. **A** Forest plot of a time-dependent Cox proportional hazards model comparing overall mortality risk between the four sepsis groups. While neither EBV reactivation alone (EBV + IP-) nor immunoparalysis alone (EBV- IP+) significantly increased mortality, patients with combined EBV reactivation and immunoparalysis (EBV + IP+) showed a 7.2-fold higher hazard of death compared with the reference group (EBV- IP-). Asterisks mark significance **B** Pairwise post-hoc contrasts confirmed that EBV + IP+ differed significantly from both EBV- IP + and EBV + IP-, indicating that the combination of viral reactivation and immune paralysis confers the highest time-dependent mortality risk in the cohort. The Cox model was based on start-stop intervals and adjusted for age and sex. The SOFA core was not included in this model, as disease severity is considered biologically intertwined with IP and part of the sepsis endotype definition rather than a confounder in this analysis. Hazard ratios (HRs) are displayed on a logarithmic scale with 95% robust confidence intervals, using EBV- IP- as the reference group. Pairwise comparisons between all group combinations were derived from the same Cox model by calculating linear contrasts of the estimated log-hazard coefficients with their corresponding robust variance-covariance matrix

Given the strong association between EBV reactivation and mortality, the time-dependent Cox model was expanded to include all four sepsis subgroups, again adjusted for age and sex. Compared with the reference group EBV- IP-, only patients with combined EBV reactivation and immunoparalysis (EBV + IP+) showed a significantly higher hazard of death (HR 7.23, 95% CI 2.24–23.3, *p* = 0.0009; Fig. 3A). Pairwise contrasts between model coefficients confirmed that EBV + IP+ carried the highest mortality risk among all groups (vs. EBV- IP+, *p* = 0.0038; vs. EBV + IP-, *p* = 0.004; Fig. 3B), consistent with a combined effect of viral reactivation and immune dysfunction on outcome.

**Fig. 4:**
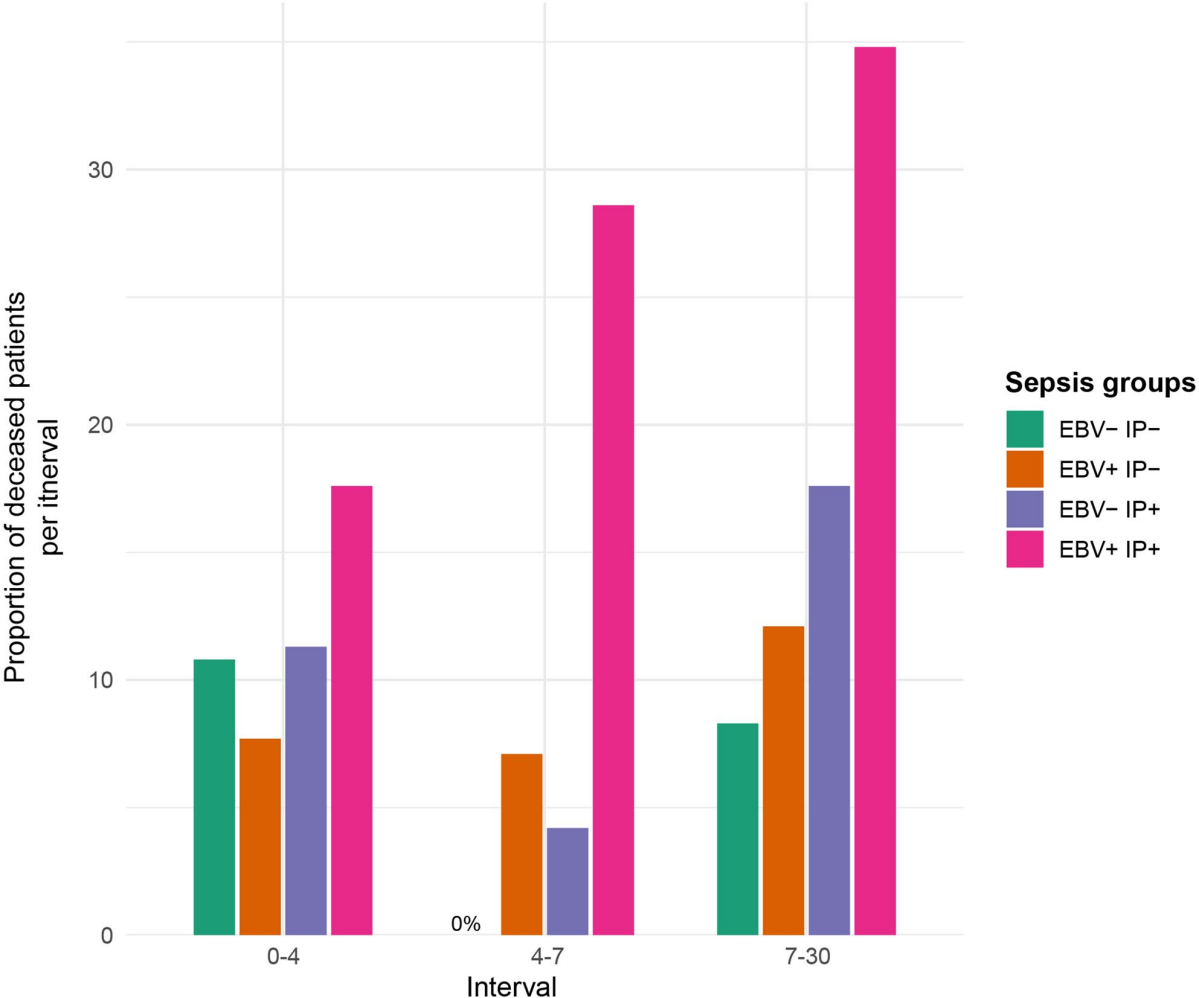
Descriptive interval-specific mortality proportions across sepsis subgroups. Shown are the crude proportions of deceased patients per sepsis group (EBV- IP-, EBV + IP-, EBV- IP+, EBV + IP+) within each observation interval (ICU days 0–4, 4–7, and 7–30). This figure provides a descriptive overview of mortality patterns over time without adjustment for differences in observation time or censoring. Group membership reflects the EBV and IP status within the respective time interval and was treated as time-varying rather than absorbing. Patients could transition between groups across intervals and were censored at death or ICU discharge. Across all intervals, the EBV + IP+ group showed the highest proportion of deaths, with an increasing trend over the course of ICU stay

This pattern was further supported by interval-specific mortality proportions providing a descriptive overview of deaths across sepsis subgroups. Here, in each interval, the EBV + IP+ group showed the highest death rates (0–4 days = 17.6%; 4–7 days = 28.6%; 7–30 days = 34.8%; Fig. 4).

**Fig. 5:**
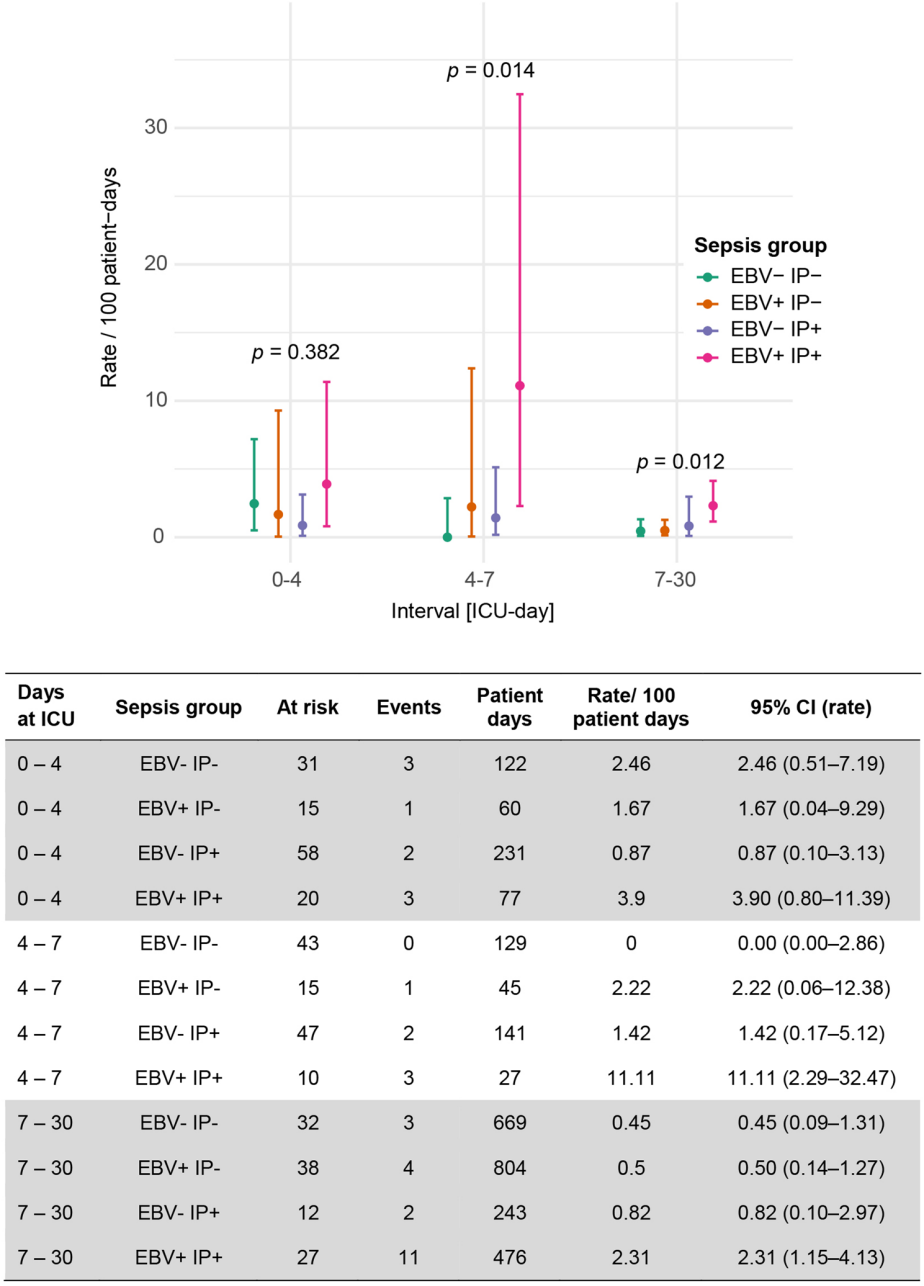
Time-standardized interval-specific mortality rates across sepsis subgroups. Shown are mortality rates per 100 patient-days (mean ± 95% CI) for the three observation intervals (ICU days 0–4, 4–7, and 7–30) across the four sepsis groups (EBV- IP-, EBV + IP-, EBV- IP+, EBV + IP+). In contrast to the descriptive proportions shown in Fig. 4, this analysis accounts for differences in observation time and censoring by calculating time-standardized mortality rates. Mortality rates were calculated by dividing the number of deaths by the total number of patient-days at risk, thereby providing a time-adjusted estimate or mortality. Rate estimates and 95% CIs were derived from Poisson regression models with exact Poisson CIs. P-values represent global between-groups differences within each interval and were obtained using likelihood ratio test (omnibus test) from the Poisson regression models. From ICU day 4 onwards, patients in the EBV + IP+ group exhibited significantly higher mortality rates compared with other subgroups. The accompanying table summarizes the number of patients at risk, deaths, cumulative observation time, and calculated rates for each group and interval

To account for differences in observation time and censoring, mortality rates per 100 patient-days were subsequently calculated, revealing significant differences during ICU days 4–7 and 7–30 (Fig. 5).

### High level of cytokine production in patients with IP and EBV reactivity

To explore biological correlates of these survival differences, plasma pro-inflammatory cytokines were quantified by flow cytometer (Legendplex^®^) together with standard laboratory parameters reflecting renal and metabolic function (measured at ICU days 0, 4, 7). Because EBV and IP status were modeled as time-varying exposures, group comparisons were performed separately within each predefined observational interval (days 0–4, 4–7, and 7–30).

Within each interval, differences between the four sepsis subgroups were first assessed using the non-parametric Kruskal-Wallis test. When a significant global difference was detected within at least one time interval, pairwise group comparisons were performed using Dunn’s post-hoc test with Holm correction for multiple testing. This interval-specific approach identified significant between-group differences in several immunological and metabolic parameters, most prominently mHLA-DR expression (*p* < 0.0001 in all intervals). In addition, IL-6, IL-8, IL-10, IL-17 A, IL-18, MCP-1, and Urea-N showed significant interval-specific differences, predominantly during the intermediate phase (days 4–7), with temporal variation in the strength and pattern of association (Table 2).

As expected based on the predefined definition of immunoparalysis (mHLA-DR < 5,000 molecules per monocyte), mHLA-DR differed significantly between IP + and IP- groups at all time points. This finding reflects subgroup allocation by design and serves as an internal validation of the classification approach. Importantly, no significant difference in mHLA-DR expression was observed between EBV- IP + and EBV + IP+ patients, indicating that the increased mortality observed in the EBV + IP+ subgroup cannot be explained by a more pronounced degree of monocyte deactivation alone. Boxplots illustrating interval-specific group distributions and significant pairwise comparisons are shown in Supplementary Fig. 1. These analyses demonstrate a consistent directionality of elevated cytokine serum concentrations in the EBV + IP+ subgroup across intervals, although not all pairwise contrasts reached statistical significance after adjustment for multiple testing.

**Table 2:** Biological and clinical characteristics associated with sepsis subgroups

Variable	*p*-value interval specific		
	[0–4]	[4–7]	[7–30]
*mHLA-DR	< 0.0001	< 0.0001	< 0.0001
*IL-6	0.128	0.001	0.041
*IL-8	0.128	0.009	0.003
*IL-10	0.041	0.013	0.166
*IL-17 A	0.208	0.024	0.316
*IL-18	0.112	0.027	0.407
*MCP-1	0.451	0.003	0.249
*Urea-N	0.514	0.0156	0.218
IL-1β	0.756	0.377	0.158
IL-12p70	0.1	0.164	0.783
IL-23	0.804	0.978	0.561
IL-33	0.456	0.978	0.337
IFNα2	0.681	0.808	0.56
IFNγ	0.758	0.471	0.646
TNFα	0.735	0.744	0.11
eGFR	0.28	0.928	0.774
Creatine	0.649	0.31	0.608
Blood pressure - diastolic	0.761	0.854	0.784
Blood pressure – systolic	0.459	0.389	0.383
Blood pressure (MAP)	0.245	0.722	0.455

Statistical comparison was performed by Kruskal-Wallis test. mHLA-DR is shown as a reference variable defining immunoparalysis and is included as an internal validation of subgroup classification. It was not interpreted as an independent biological correlate. Variables with significant *p*-values (< 0.05) are marked by asterisks

To identify variables that most distinctly separated the four sepsis subgroups, group-wise mean ranks were compared as an exploratory descriptive approach (Table 3).

**Table 3:** Pairwise comparison of min and max values of sepsis group defining variables

Variable	*p*-val.	Group [min]	*n* [min]	median [min]	IQR [min]	Group [max]	*n* [max]	median [max]	IQR [max]	fc	Δ rank
mHLA-DR	< 0.0001	EBV + IP+	57	2778.0	2104.9–3557.0	EBV- IP-	89	8959.0	6826.66-12222.39	3.23	166
IL-6	< 0.0001	EBV + IP-	60	35.3	17.41–99.25	EBV- IP+	104	174.0	57.02-352.39	4.93	73.3
IL-8	< 0.0001	EBV + IP-	60	39.9	22.52–85.95	EBV + IP+	55	104.0	47.39-207.32	2.6	75
IL-10	0.0003	EBV- IP-	82	3.25	0–10	EBV + IP+	55	13.5	4.94–28.05	4.16	62.5
MCP-1	0.0003	EBV- IP-	82	180.0	117.87 -311.92	EBV + IP+	55	276.0	164.56-574.78	1.53	46.4
Urea-N	0.0046	EBV- IP-	46	24.0	15.8–29.87	EBV + IP+	28	33.4	27.35–38.54	1.39	30.8
IL-17 A	0.0076	EBV + IP-	60	0.445	0–0.72	EBV + IP+	55	0.73	0.46–1.1	1.64	54.3
IL-18	0.0089	EBV- IP-	82	226.0	120.74 -490.28	EBV + IP+	55	503.0	210.86-1265.69	2.23	48.1

Group-wise rank distributions of significant markers were compared across the four sepsis groups using Kruskal-Wallis tests. For each variable, group-specific median concentrations with interquartile ranges (IQR) and corresponding sample sizes are reported. The groups with the highest and lowest mean ranks (based on pooled ranks) were identified, and the rank-difference (max - min) is shown as an exploratory measure of discriminatory separation. Fold-change (fc) represents the ratio of medians between the highest and lowest groups

The most pronounced separation was observed for mHLA-DR (*p* < 0.0001), which showed highest ranks in EBV- IP- and lowest in EBV + IP+, indicating marked down-regulation of mHLA-DR in patients with EBV reactivation. Conversely, IL-8 (Δrank 75; *p* < 0.0001), IL-10 (Δrank 62.5; *p* = 0.0003), MCP-1 (Δrank 46.4; *p* = 0.0003), IL-17 A (Δrank 54.3; *p* = 0.0076), IL-18 (Δrank 48.1; *p* = 0.0089), and Urea-N (Δrank 30.8; *p* = 0.0046) showed the opposite trend, with highest ranks in EBV + IP+ and lowest in EBV- IP- or EBV + IP-. Interestingly, IL-6 showed the highest group ranks in EBV- IP+ patients (*p* < 0.0001). The largest rank difference was found for mHLA-DR (Δrank = 166), followed by IL-6 and IL-8 (Δrank ≈ 73–75), highlighting a strong divergence between inflammatory activation and immune suppression. These findings were consistent with the time-interval dependent upward trend of serum cytokine concentrations in EBV + IP+ patients shown in Supplementary Fig. 1.

**Fig. 6:**
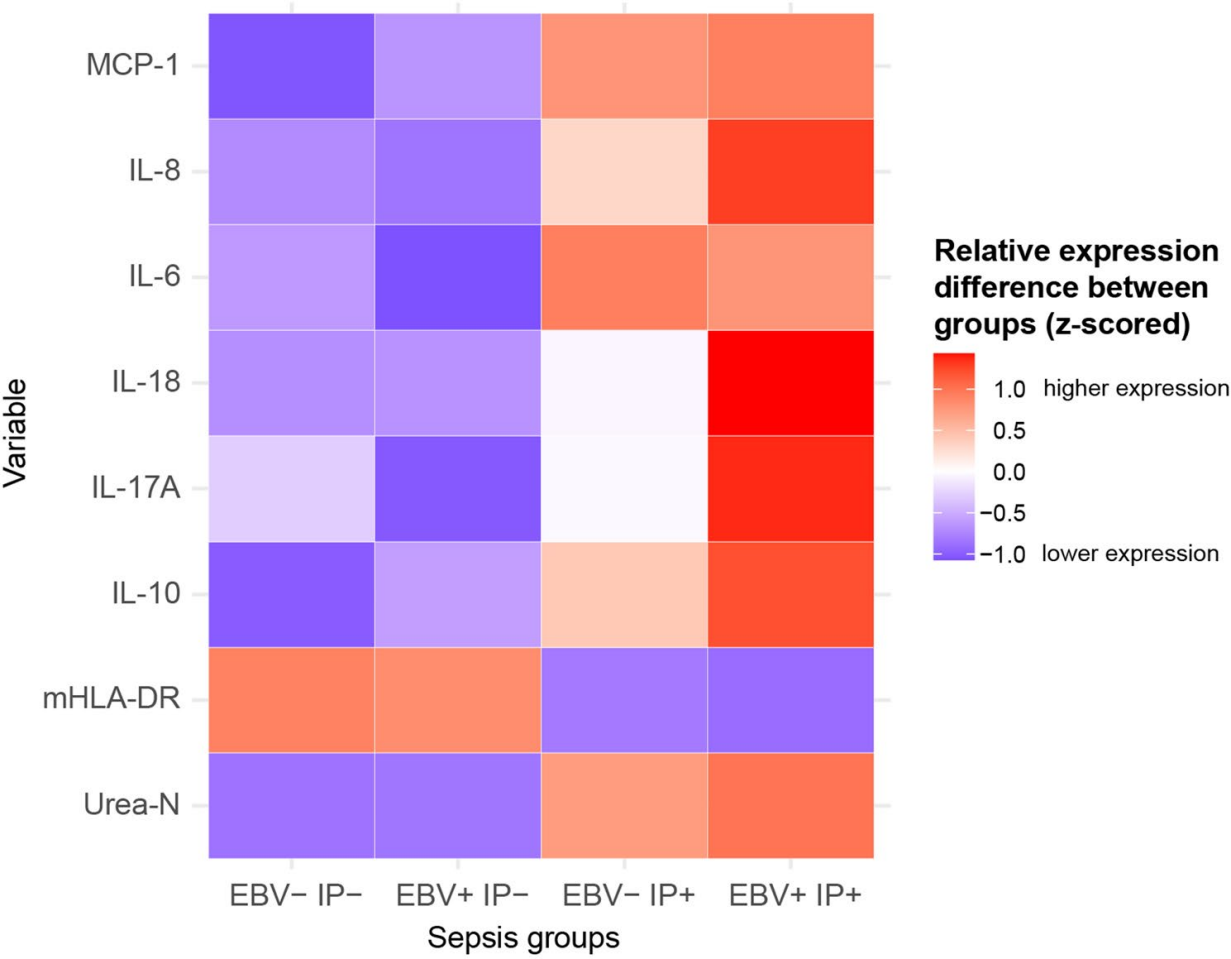
Rank-based group profiles of immunological and renal function parameters in sepsis groups. Heatmap visualization of the rank-based analysis summarized in Table 3. Z-standardized mean ranks are shown for eight significant variables across the four sepsis groups. For each variable, pooled ranks were computed across all observation, averaged per group, and subsequently z-transformed per variable (mean = 0, SD = 1) to highlight relative differences between groups while ensuring comparability across markers with heterogeneous scales. Warm colors indicate above-average mean ranks (relatively higher expression/concentration levels), whereas cool colors indicate below-average mean ranks (lower expression/concentration levels) relative to the overall distribution of that variable. Values therefore reflect relative, not absolute, expression or concentration levels. The plot highlights the inverse pattern of mHLA-DR (lowest in EBV + IP+) compared with the cytokine cluster and urea nitrogen, which peak in EBV + IP+, reflecting a hyperinflammatory yet immunosuppressed phenotype

A rank-based heatmap displaying the z-standardized mean ranks of the eight variables across the four sepsis groups highlights a distinct contrast between reduced mHLA-DR expression and elevated cytokine activity (IL-6, IL-8, IL-10, IL-17 A, IL-18, MCP-1) in EBV + IP+ patients (Fig. 6A). A radar plot (Supplementary Fig. 2) of normalized mean ranks (scaled 0–1) provides and integrative overview of each group’s immune signature. The EBV + IP+ subgroup exhibits a combined hyperinflammatory yet immunosuppressed profile, characterized by maximal cytokine activity but minimal mHLA-DR expression; EBV- IP+ displays a moderately inflammatory phenotype dominated by IL-6 elevation; EBV + IP- shows a balanced cytokine profile; and EBV- IP- represents the most “immunocompetent” baseline phenotype.

**Fig. 7:**
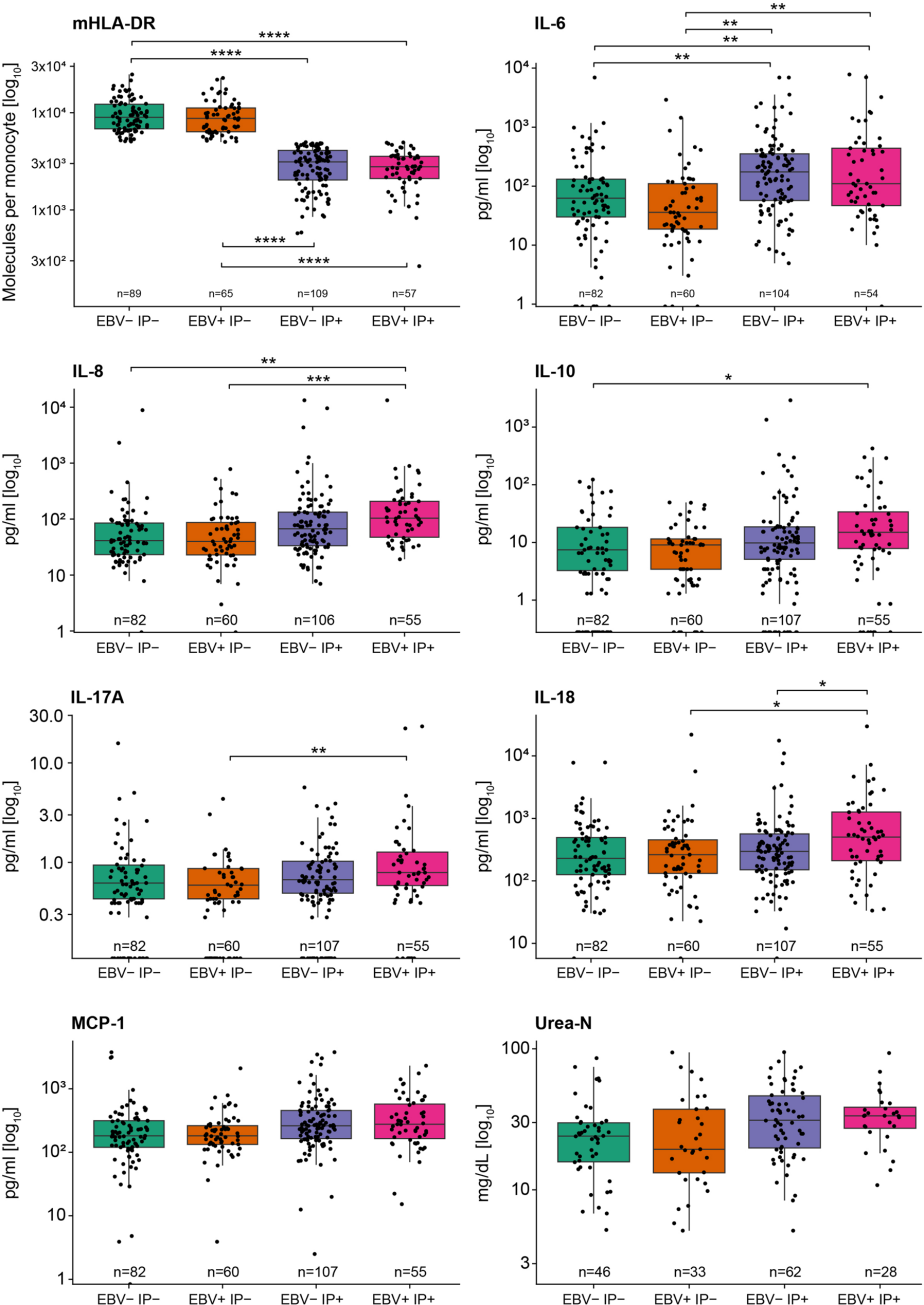
Distribution of key immunological and renal/metabolic markers across sepsis subgroups. Boxplots display all individual measurements of the eight markers identified as discriminatory between sepsis groups (Table 2), including immunological and metabolic/renal parameters. Each dot represents a single sample measurement, and the total n sample size is indicated above y-axes. Group differences were analyzed using mixed-effects models with log_10_-transformed marker values as the dependent variable, sepsis group and visit as fixed effects, and a random intercept for patient ID to account for repeated measurements within individuals. Pairwise comparisons were performed using estimated marginal means with Holm adjustment for multiple testing. Asterisks indicate Holm-adjusted *p*-values from pairwise comparisons of estimated marginal means derived from the mixed-effects model (*p < 0.05, **p < 0.01, ***p < 0.001, ****p < 0.0001)

Finally, to visualize all individual measurements across the ICU course independent of internal stratification, key marker concentrations were analyzed using linear mixed-effects models accounting for repeated measurements within patients (Fig. 7). For each marker, log_10_-transformed values were modeled as a function of sepsis group and visit, including a random intercept for patient ID to account for intra-individual dependency. The mixed model analysis confirmed significantly overall group effects for mHLA-DR and all major cytokines identified in the interval-specific analyses (Supplementary Fig. 1). As expected, mHLA-DR levels were significantly lower in both IP+ groups compared with IP- groups. Although the rank-based comparison (Table 3) suggested the lowest mHLA-DR expression in EBV + IP+, mixed model analysis revealed no significant difference between EBV- IP + and EBV + IP+, confirming comparable monocyte deactivation irrespective of EBV status. In contrast, cytokine concentrations of IL-6, IL-8, IL-10, IL-17 A, IL-18, MCP-1 showed at least a consistent upward trend in EBV + IP+. Interestingly, IL-6 showed as significant upregulation in both IP+ groups compared to IP- irrespectively of EBV status. Notably, IL-18 remained significantly higher in EBV + IP+ even when compared with EBV- IP+. Comparing IL-17A in both EBV+ groups showed a significantly higher expression in combination with an immunoparalysis.

These findings indicate that EBV reactivation in the presence of immunoparalysis is associated with an amplified cytokine response. Even after accounting for intra-individual clustering of repeated measurements, EBV + IP+ patients exhibit consistently higher cytokine levels, indicating a true biological amplification rather than a statistical artefact.

Overall, this final analysis confirms that while immunoparalysis (low mHLA-DR) defines a shared suppressed state across both IP+ groups, EBV reactivation adds an additional inflammatory component, resulting in the strongest cytokine activation and worst prognosis in the EBV + IP+ subgroup.

## Discussion

In this study, we demonstrate that Epstein-Barr virus (EBV) reactivation and sepsis-induced immunoparalysis (IP) act in concert to define a distinct immune endotype in critically ill sepsis patients, characterized by concurrent hyperinflammation, profound monocyte deactivation, and excess mortality. The higher mortality was consistently seen both in the simple comparison of death rates within each time interval and in analyses that accounted for different ICU length of stay. This suggest that the association is real and time-dependent, rather than being explained by unequal follow-up times between groups.

Using a time-dependent Cox model, we show that EBV positivity was independently associated with a more than threefold increase in mortality risk, and that patients exhibiting both EBV reactivation and IP had the highest hazard of death (HR 7.2). While EBV positivity remained independently associated with mortality after adjustment for disease severity, the association between IP and outcome was attenuated upon inclusion of the SOFA score. This finding suggests that IP is closely linked to overall disease severity and may partly reflect downstream immune dysfunction in patients with advanced organ failure.

This combination phenotype (EBV + IP+) was further marked by strong cytokine upregulation – particularly IL-6, IL-8, IL-10, IL-17 A, IL-18, and MCP-1 – reflecting a paradoxical state of simultaneous immune suppression and hyperactivation. Together, these findings highlight the dynamic interaction between viral reactivation and immune dysfunction during the septic trajectory and suggest that EBV may act as a secondary amplifier of inflammation in immunocompromised hosts.

EBV reactivation is increasingly recognized as a marker of immune dysregulation in critically ill patients. Previous studies have reported EBV DNAemia in 35–70% of ICU patients with sepsis, often correlating with longer ICU stays, increased secondary infections, and higher mortality [14–16]. Notably, Guiouillier et al. [29] recently reported that EBV reactivation at ICU admission was associated with increased mortality compared to both patients without reactivation and those with later reactivation. However, their analysis relied primarily on Kaplan-Meier survival curves based on baseline EBV status in a mixed ICU cohort that was not restricted to sepsis. Such an approach does not account for the time-dependent nature of viral reactivation, nor does it adjust for evolving disease severity, thereby potentially introducing immortal-time bias and residual confounding. Our study extends these findings by applying a time-dependent approach, which accounts for changing EBV and IP status over multiple intervals. By modeling risk across distinct time window (0–4, 4–7, 7–30), we demonstrate that EBV positivity is not merely a static marker of severe disease but a dynamic process associated with rising mortality risk over time. This temporal perspective avoids immortal-time bias inherent in classical Kaplan-Meier analyses, and allows adjustment for baseline severity (SOFA score) and demographic factors, providing a more accurate estimation of risk.

Immunoparalysis, reflected by low monocyte HLA-DR expression, is a well-established hallmark of sepsis-induced immune dysfunction [30, 31]. It results from sustained exposure to inflammatory mediators such as IL-6, IL-10, and corticosteroids, leading to impaired antigen presentation and decreased responsiveness to pathogens [9, 32]. In this state, the reactivation of latent viruses, particularly EBV and CMV, is thought to result from inadequate cytotoxic control by exhausted T and NK cells [33–35]. Our data support this concept: patients with low mHLA-DR levels (IP+) exhibited higher rates of EBV reactivation and poorer survival. However, this interaction appears bidirectional. Once reactivated, EBV may itself aggravate systemic inflammation through activation of NF-κB, TLRs, and inflammasome pathways, further amplifying cytokine release [35, 36]. This feed-forward loop between immune suppression and viral activation could explain the pronounced cytokine storm in EBV + IP+ patients observed in our cohort.

The EBV + IP+ subgroup exhibited a distinct immunological profile compared with other sepsis phenotypes. While both IP+ groups shared reduced mHLA-DR expression, EBV + IP+ patients consistently demonstrated higher concentrations of multiple cytokines (IL-6, IL-8, IL-10, IL-17A, IL-18, MCP-1). Importantly, mixed-effects modeling confirmed that IL-18 levels were significantly higher in EBV + IP+ compared with EBV- IP+ patients, whereas other cytokines showed a consistent upward trend without reaching statistical significance in this direct comparison. These findings indicate that EBV reactivation in the context of immunoparalysis is associated with an amplified inflammatory signature, even when accounting for repeated measurements and intra-individual dependency.

This pattern points to a mixed immune state, simultaneously hyperinflammatory and immunosuppressed, reminiscent of the “Mixed Antagonist Response Syndrome” (MARS) previously described in sepsis [23, 24]. The indicated higher expression of IL-8 together with the significantly elevated levels of IL-18 in EBV + IP+ than in EBV- IP+ patients, is consistent with reported EBV-induced inflammasome activation and neutrophil recruitment [20–22]. The concurrent increases in IL-10 and IL-17A may represent a compensatory anti-inflammatory feedback attempting to restrain excessive immune activation [2, 37, 38]. Altogether, this constellation of elevated cytokines with low monocyte activation defines an EBV-driven immune endotype with poor prognosis.

From a translational standpoint, these results have several implications. First, the combination of EBV qPCR and mHLA-DR monitoring could serve as a dynamic biomarker panel for risk stratification in sepsis. Unlike static measurements, repeated assessment of both viral load and immune competence allows for temporal resolution of immune recovery of collapse. Second, identifying patients with the EBV + IP+ signature could guide immunomodulatory therapy. Prior trials have demonstrated survival benefits from IFNγ or GM-CSF in patients with low mHLA-DR [30]; however, EBV reactivation status was not considered. Our data suggest that antiviral and immune-stimulatory strategies may need to be combined in this subgroup. In this context, recent evidence has highlighted the EBV-encoded deoxyuridine triphosphate nucleotidohydrolase (dUTPase) as an early viral activity marker with intrinsic pro-inflammatory properties, detectable even before measurable DNAemia [39–43]. Given the ability of EBV dUTPase to activate TLR2/NF-κB and drive early cytokine release, measuring dUTPase, either directly or via neutralizing antibodies, may flag incipient EBV reactivation before qPCR positivity. In high risk EBV + IP+ phenotypes, such an “early EBV” marker could enable earlier risk stratification and timely immunomodulatory / antiviral interventions. However, prospective validation studies in sepsis are required. Finally, the presence of EBV reactivation should not be dismissed as an epiphenomenon, it may signal ongoing immune failure requiring targeted intervention.

Several limitations of this study should be acknowledged. First, this multi-center study is partially of retreospective character. EBV reactivation and immunoparalysis were modeled as time-dependent exposures, resulting in dynamic group membership during analyzed time intervals rather than fixed baseline cohorts. Consequently, subgroup sizes varied across time intervals and should be interpreted in the context of the corresponding risk sets. To improve transparency, the number of patients at risk and events per group and time interval is reported in the table of Fig. 5.

Although the number of patients contributing risk time to each group was substantial, the precision of some subgroup comparisons was limited by the number of events (deaths) occurring within individual categories. In particular, contrasts between EBV + IP- and EBV- IP+ groups were characterized by wide confidence intervals, indicating limited ability to exclude moderate hazard differences. Accordingly, non-significant findings for these comparisons should not be interpreted as evidence of equivalence.

Cytokine measurements were performed at predefined time points rather than continuously, potentially missing transient fluctuations. While proportional hazard assumptions were verified, time-dependent modeling relies on interval accuracy and may underestimate rapid changes in viral or immune states. Finally, although EBV reactivation, particularly in combination with immunoparalysis, was strongly associated with mortality, the observational design precludes causal inference. EBV may act either as a driver amplifying immune dysfunction or as a marker identifying patients in whom immune control has already collapsed.

In addition, while cytokine concentrations were directionally higher in the EBV + IP+ subgroup across multiple analyses, not all pairwise comparisons reached statistical significance. This is partly attributable to limited effective sample sizes within specific interval-by-group strata, as patients could change EBV/IP status over time and not all biomarkers were available at each sampling point, resulting in relatively small numbers of observations in certain comparisons. Furthermore, we applied statistically conservative approaches, interval-specific Kruskal-Wallis tests followed by Dunn’s post-hoc comparisons with Holm adjustment (Table 2, Supplementary Fig. 1), as well as linear mixed-effects models with Holm-adjusted estimated marginal means contrasts to account for repeated measurements (Fig. 7). Together, stratification, biological variability of cytokine responses in sepsis, and multiplicity adjustment may have reduced power to detect moderate effect sizes. Therefore, the absence of statistical significance in some comparisons should not be equated with absence of biological relevance, particularly given the consistent directionality of cytokine elevations observed in EBV + IP+ patients.

Importantly, the present study is also limited by the lack of experimental validation. While elevated cytokine levels were observed in the EBV + IP+ group, the underlying molecular mechanisms by which EBV reactivation may aggravate immunoparalysis cannot be directly inferred from our data. The proposed links between EBV reactivation, inflammatory signaling, and monocyte deactivation are therefore based on prior experimental and translational studies and should be regarded as hypothesis-generating rather than causal. Future studies combining longitudinal clinical sampling with in vitro and in vivo functional models will be required to delineate the mechanistic interplay between latent viral reactivation and immune failure in sepsis.

Although no significant association were observed between antiviral or immunomodulatory therapy and EBV or immunoparalysis status, the number of exposed patients was limited.

In summary, despite these limitations, our study identifies a high-risk sepsis endotype defined by concurrent EBV reactivation and immunoparalysis. This EBV + IP+ phenotype combines extreme cytokine activation with severe monocyte deactivation, resulting in the highest mortality among all subgroups. By integrating viral and host immune dynamics into a time-dependent framework, we provide a novel perspective on how latent viral reactivation intersects with immune failure and critical illness. Future prospective studies, as mentioned, are warranted to validate these findings and to explore whether early detection of EBV + IP+ signature can inform individualized immunotherapeutic strategies. Ultimately, understanding the EBV-immunoparalysis axis may open new avenues for precision immunomonitoring and targeted interventions in sepsis.

## Supplementary Information

Below is the link to the electronic supplementary material.

Supplementary Material 1.

Supplementary Material 2: Interval specific distribution of key immunological and biochemical markers across sepsis groups and time intervals. Boxplots depict the distribution of selected cytokines, mHLA-DR expression, and renal parameters across the four sepsis groups within each predefined ICU interval (days 0–4, 4–7, 7–30). Group comparisons were performed separately within each interval using the non-parametric Kruskal-Wallis test, accounting for the time-varying nature of EBV and IP status. Furthermore, pairwise comparisons were conducted using Dunn’s post-hoc test with Holm adjustment for multiple testing. Asterisks indicate adjusted p-values from post-hoc comparisons (*p < 0.05, **p < 0.01, ***p < 0.001, ****p < 0.0001). Only variables with at least one interval-specific global p-value < 0.05 are shown. All values are displayed on a log10 scale.

Supplementary Material 3: Radar plot of rank-based immune profiles across sepsis groups. Radar plot showing normalized mean ranks (min-max scaled 0–1) derived from the rank-based analysis presented in Table 3. Each axis represents one marker, and values reflect the relative rank-based contribution of that marker within each group. Scaling was performed per variable to allow cross-marker comparison independent of absolute concentration ranges. The EBV + IP+ group exhibits the most pronounced combined hyperinflammatory and immunosuppressed profile, whereas EBV- IP- displays features consistent with a baseline “immunocompetent” phenotype. This visualization is intended for descriptive subgroup profiling.

## Data Availability

The datasets used and/or analyzed during this study are available from the corresponding author upon reasonable request.
