# Supplementary material for: EBV reactivation and immunoparalysis indicate a harmful immune endotype in sepsis
Source: Crit Care. 2026 May 7;30:242. doi: 10.1186/s13054-026-05966-2 (PMC13154855; doi:10.1186/s13054-026-05966-2)
Supplement: Supplementary file 1 — Supplementary Material 1. [file 13054_2026_5966_MOESM1_ESM.docx]

**Supplementary Table 1: Association between antiviral / immunomodulatory therapy and EBV/IP status**

| **Analysis** | **Outcome A**  **IP- / EBV-** | **Outcome B**  **IP+ / EBV+** | **OR (95% CI)** | ***p*-val.** |
| --- | --- | --- | --- | --- |
|  | **Exposure No / Exposure Yes** | **Exposure No / Exposure Yes** |  |  |
| Pre-baseline IM  ↔  Baseline IP | 35 (76.1%) / 11 (23.9%) | 64 (82.1%) / 14 (17.9%) | 0.70 (0.26–1.90) | 0.489 |
| Pre-baseline AV  ↔  Baseline EBV | 80 (89.9%) / 9 (10.1%) | 31 (88.6%) / 4 (11.4%) | 1.15 (0.24–4.49) | >0.99 |
| ICU-post IM  ↔  Later IP | 29 (87.9%) / 4 (12.1%) | 10 (76.9%) / 3 (23.1%) | 2.13 (0.27–15.20) | 0.385 |
| ICU-post AV  ↔  Later EBV | 29 (96.7%) / 1 (3.3%) | 57 (96.6%) / 2 (3.4%) | 1.02 (0.05–62.05) | >0.99 |

The table summarizes analyses evaluating whether prior (pre-baseline) or ICU-administered antiviral (AV) or immunomodulatory (IM) therapy was associated with immunoparalysis (IP) or EBV positivity at baseline or during ICU follow-up. For each comparison, absolute numbers and percentages of exposed patients (No / Yes) are shown separately for those without and with (Outcome A; Outcome B) for the respective condition (baseline IP or EBV positivity; later IP or EBV development). Odds ratios (OR) with 95% confidence (CI) and p-values were calculated using Fisher's exact test. No statistically significant associations were observed.
