# Supplementary figures and images for: EBV reactivation and immunoparalysis indicate a harmful immune endotype in sepsis
Source: Crit Care. 2026 May 7;30:242. doi: 10.1186/s13054-026-05966-2 (PMC13154855; doi:10.1186/s13054-026-05966-2)

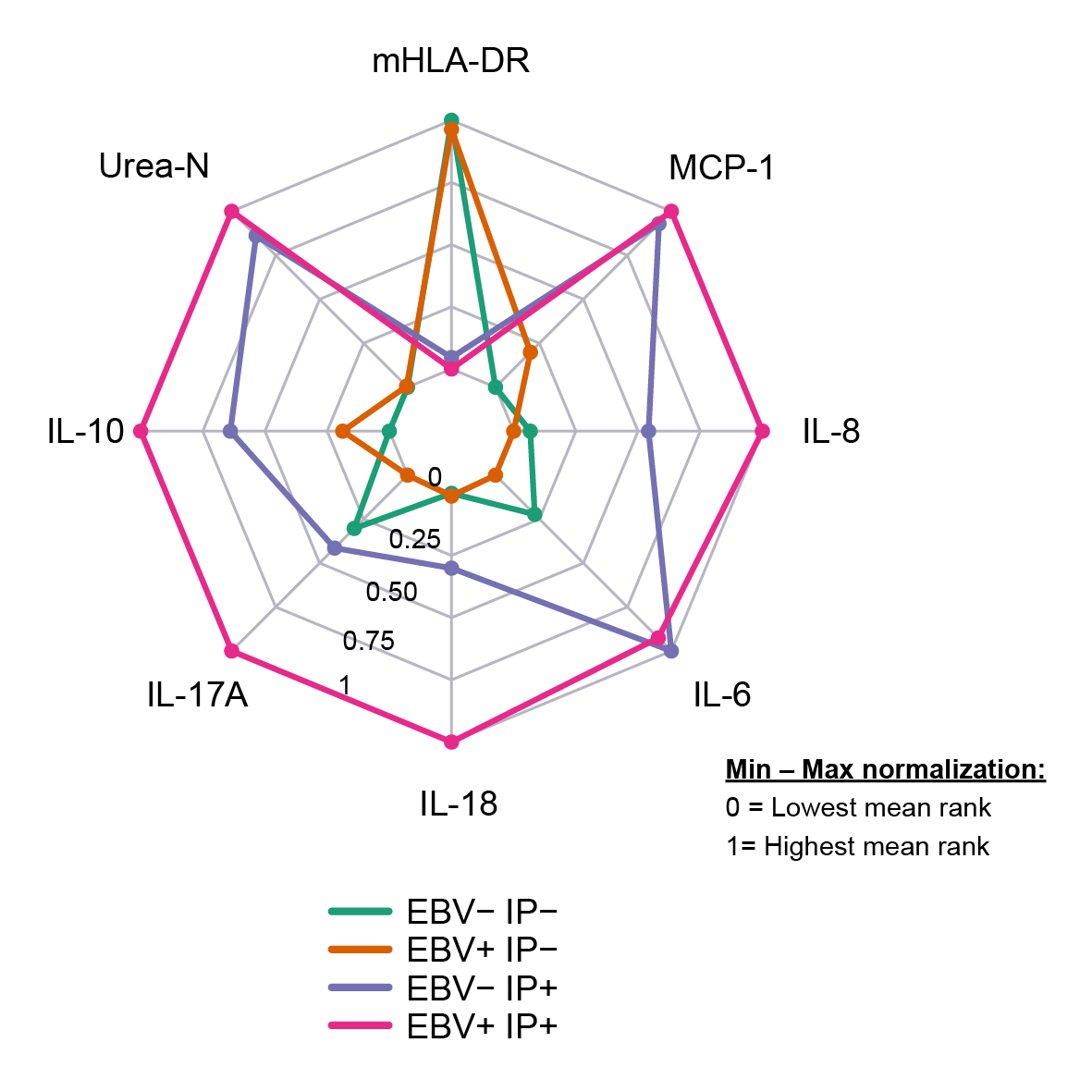

Supplement: Supplementary file 2 — Supplementary Material 2: Interval specific distribution of key immunological and biochemical markers across sepsis groups and time intervals. Boxplots depict the distribution of selected cytokines, mHLA-DR expression, and renal parameters across the four sepsis groups within each predefined ICU interval (days 0–4, 4–7, 7–30). Group comparisons were performed separately within each interval using the non-parametric Kruskal-Wallis test, accounting for the time-varying nature of EBV and IP status. Furthermore, pairwise comparisons were conducted using Dunn’s post-hoc test with Holm adjustment for multiple testing. Asterisks indicate adjusted p-values from post-hoc comparisons (*p < 0.05, **p < 0.01, ***p < 0.001, ****p < 0.0001). Only variables with at least one interval-specific global p-value < 0.05 are shown. All values are displayed on a log10 scale. [file 13054_2026_5966_MOESM2_ESM.jpg]

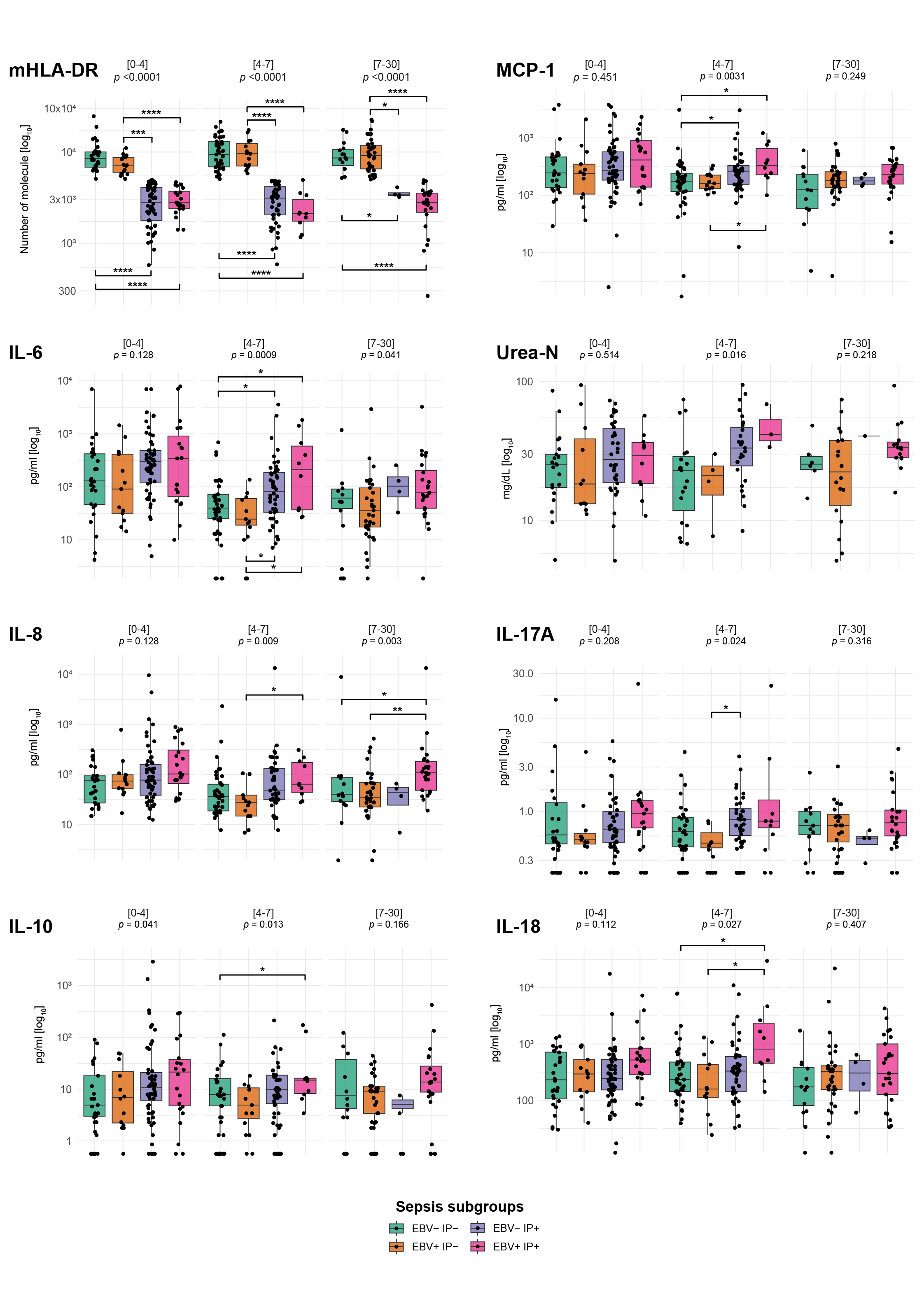

Supplement: Supplementary file 3 — Supplementary Material 3: Radar plot of rank-based immune profiles across sepsis groups. Radar plot showing normalized mean ranks (min-max scaled 0–1) derived from the rank-based analysis presented in Table 3. Each axis represents one marker, and values reflect the relative rank-based contribution of that marker within each group. Scaling was performed per variable to allow cross-marker comparison independent of absolute concentration ranges. The EBV + IP+ group exhibits the most pronounced combined hyperinflammatory and immunosuppressed profile, whereas EBV- IP- displays features consistent with a baseline “immunocompetent” phenotype. This visualization is intended for descriptive subgroup profiling. [file 13054_2026_5966_MOESM3_ESM.jpg]
